# Fatal Sickle Cell Disease and Zika Virus Infection in Girl from Colombia

**DOI:** 10.3201/eid2205.151934

**Published:** 2016-05

**Authors:** Laura Arzuza-Ortega, Arnulfo Polo, Giamina Pérez-Tatis, Humberto López-García, Edgar Parra, Lissethe C. Pardo-Herrera, Angélica M. Rico-Turca, Wilmer Villamil-Gómez, Alfonso J. Rodríguez-Morales

**Affiliations:** Entidades Promotoras de Salud Barrios Unido Mutual, Quibdó, Colombia (L. Arzuza-Ortega);; Empresa Social del Estado Hospital de Malambo, Malambo, Colombia (A. Polo);; Hospital Metropolitano, Barranquilla, Colombia (G. Pérez-Tatis, H. López-García);; Instituto Nacional de Salud, Bogotá, Colombia (E. Parra, L.C. Pardo-Herrera, A.M. Rico-Turca);; Hospital Universitario de Sincelejo, Sincelejo, Colombia, (W. Villamil-Gómez);; Universidad del Atlántico, Barranquilla (W. Villamil-Gómez);; Universidad de Cartagena, Cartagena, Colombia (W. Villamil-Gómez);; Universidad Tecnológica de Pereira, Pereira, Colombia (A.J. Rodriguez-Morales)

**Keywords:** Zika virus, ZIKV, viruses, sickle cell disease, hemoglobin, Colombia

**To the Editor:** Zika virus, a mosquito-borne flavivirus, causes, a usually self-limiting febrile and exanthematic arthralgia syndrome that resembles dengue and chikungunya (*1*). This arboviral disease has emerged in tropical areas of Latin America, particularly in Brazil and Colombia (*2*), as a public health threat in 2015 and has spread into areas to which dengue virus (DENV) and chikungunya virus (CHIKV) are endemic (*1*–*4*).

Cases of severe and fatal Zika virus infection have not been described (*5*), and the spectrum of clinical disease remains uncertain in the setting of rapidly evolving epidemics of this arbovirus in Latin America (*1*). We report a person with sickle cell disease who acquired a Zika virus infection and died.

The patient was a 15-year-old girl who in October 2015 came to the outpatient clinic of the Hospital of Malambo (a primary-level public hospital) in Malambo (Atlántico Department) in northern Colombia. In this region, during September 22, 2015–January 2, 2016, a total of 468 suspected cases of Zika virus infection and 4 reverse transcription PCR (RT-PCR)–confirmed cases have been reported. This patient had a high fever (temperature >40°C), arthralgias, retro-ocular pain, abdominal pain, myalgias, and jaundice for the previous 4 days. She had sickle cell disease for 5 years (hemoglobin genotype SC identified by DNA analysis), but no previous hospitalizations or episodes of vaso-occlusive crises. She had never had dengue, chikungunya, or acute chest syndrome.

At admission to the hospital, the patient had a pulse rate of 112 beats/min, a respiratory rate of 24 breaths/min, a blood pressure of 110/70 mm Hg, and a temperature of 39.0°C. She had abdominal pain, no petechiae, and no lymphadenopathy. The patient was given acetaminophen. Results of a neurologic assessment were unremarkable. Clinical laboratory findings are shown in the Table.

Given these manifestations, she was given a diagnosis of a DENV infection and referred to Barranquilla Hospital Metropolitano (Barranquilla, Colombia) where she was admitted 1 day later. Physical examination showed a pulse rate of 122 beats/min, a respiratory rate of 34/min (peripheral capillary oxygen saturation 93%), a blood pressure of 112/58 mm, and a temperature of 37.5°C. She had generalized jaundice, respiratory distress, severe abdominal pain, hepatomegaly, and splenomegaly, but no lymphadenopathy. The patient was conscious (stuporous) and had a Glasgow Coma Scale score of 13. Cardiovascular assessment showed tachycardia and a holosystolic murmur (grade II) but no other findings. 

The patient was then transferred to the pediatric intensive care unit, where she was intubated and mechanical ventilation was initiated. Her condition was considered life threatening; the patient had severe acute respiratory distress syndrome and progressive hypoxemia despite ventilator treatment, and laboratory findings worsened (Table).

**Table:** Clinical laboratory results for 15-year-old girl with sickle cell disease who died of Zika virus infection, Colombia*

Laboratory test	Baseline value	Value at hospitalization (Malambo)	Value 24 h later (ICU, Barranquilla)
Leukocyte count, × 10^9^ cells/L	10.00	8.23	ND
Hemoglobin level, g/dL	7.00	8.10	4.20
Hematocrit, %	28.00	25.00	13.00
MCV, fL/erythrocyte	73.00	73.00	ND
Reticulocytes, %	1.00	1.00	ND
Total bilirubin, mg/dL	ND	2.97	ND
Direct bilirubin, mg/dL	ND	1.67	ND
Platelet count/mL	ND	54,000.00	76,000.00
PT, s	ND	ND	33.3 (control 13.10)
aPTT, s	ND	ND	45.0 (control 29.80)
ALT, mg/dL	ND	ND	2,245.00
AST, mg/dL	ND	ND	3,215.00
LDH , IU/L	ND	ND	441.00
Alkaline hemoglobin electrophoresis, %			
HbS	ND	ND	62.50
HbC–E	ND	ND	37.50
HbF	ND	ND	0.00
Malaria thick and thin blood smears†	Not done	Not done	–
HIV-1 and HIV-2 ELISA†	Not done	Not done	–
MAT for *Leptospira* spp.†	Not done	Not done	–
RT-PCR for DENV†	Not done	Not done	–
RT-PCR for CHIKV†	Not done	Not done	–
RT-PCR for YFV†	Not done	Not done	–
RT-PCR for ZIKV†	Not done	Not done	+

*ICU, intensive care unit; ND, not determined; MCV, mean corpuscular volume; PT, prothrombin time; aPTT, activated partial thromboplastin time; ALT, alanine aminotransferase; AST, aspartate aminotransferase; LDH, lactate dehydrogenase; Hb, hemoglobin; –, negative; MAT; microscopic agglutination test; RT-PCR, reverse transcription PCR; DENV, dengue virus; CHIKV, chikungunya virus; YFV, yellow fever virus; ZIKV, Zika virus; +, positive. †Blood samples were obtained 5 days after illness onset. These tests were performed at the National Reference Laboratory of the National Institute of Health, Bogotá, Colombia.

The patient was given transfusions of blood products for treatment of anemia and thrombocytopenia. Chest radiograph and ultrasound showed an extensive right-side hemothorax. The result of a Zika virus–specific real-time RT-PCR was positive (Table). Her clinical condition deteriorated. Despite intensive treatment, the patient did not recover and died 37 hours later. An autopsy showed hepatic panacinar necrosis, erythrophagocytosis of Kupffer cells, and severe decrease of splenic lymphoid tissue (functional asplenia) with multiple drepanocytes and splenic sequestration, but no signs of yellow fever or malaria (Technical Appendix Figure).

Although sickle cell disorders are not common in Colombia, their frequency is higher along the Caribbean coast (including Atlántico Department) and 2 times that of the rest of Colombia) (*6*). Although chronic diseases, such as sickle cell disorders, are considered to be a risk factor for development of severe dengue and chikungunya (*7*,*8*), no cases have been reported in association with Zika. Reports of patients co-infected with DENV and CHIKV are rare, few details are available, and mostly restricted to few fatal cases of dengue (*9*). In patients with dengue, deaths might be higher among those who have a hemoglobin SC genotype, as recently reported (*10*). Onset of vaso-occlusion in persons with sickle cell disorders is often triggered by inflammation, as has been reported in DENV infections and which probably occurred in our patient (*8*). This complication and severe splenic sequestration, detected by autopsy, probably caused her death.

In summary, this case indicates that patients with sickle cell disorders and suspected arboviral infections should be closely monitored. Given current epidemics of Zika virus infection in Colombia (746 RT-PCR–confirmed cases and 11,712 suspected cases during September 22, 2015–January 2, 2016), atypical and severe manifestations and concurrent conditions in patients should be assessed to prevent additional deaths (*2*).

**Technical Appendix Figure.** Autopsy findings for liver and spleen of a 15-year-old girl with sickle cell disease who died of Zika virus infection, Colombia.
