# Supplementary material for: Fatal Sickle Cell Disease and Zika Virus Infection in Girl from Colombia
Source: Emerg Infect Dis. 2016 May;22(5):925–7. doi: 10.3201/eid2205.151934 (PMC4861530; doi:10.3201/eid2205.151934)
Supplement: Supplementary file 1 — Technical Appendix Figure. Autopsy findings for liver and spleen of a 15-year-old girl with sickle cell disease who died of Zika virus infection, Colombia. [file 15-1934-Techapp-s1.pdf]

Article DOI: http://dx.doi.org/10.3201/eid2205.151934

# Fatal Zika Virus Infection in Patient with Sickle Cell Disease, Colombia

## Technical Appendix

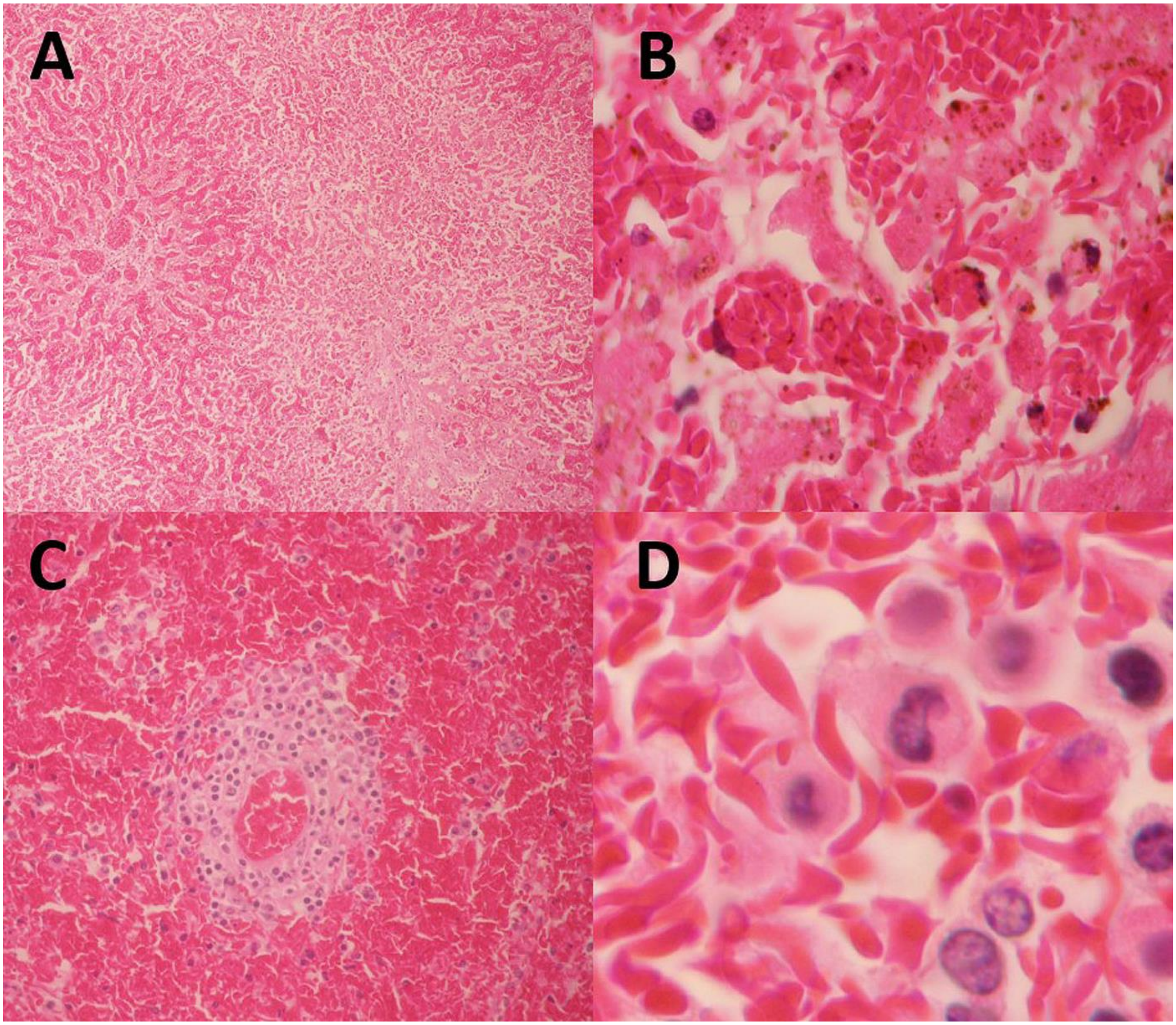


**Technical Appendix Figure.** Autopsy findings for liver and spleen of a 15-year-old girl with sickle cell disease who died of Zika virus infection, Colombia. A) Liver showing panacinar necrosis. B) Liver showing erythrophagocytosis of Kupffer cells. C) Spleen showing severe decrease of white pulp (functional asplenia). D) Spleen showing multiple splenic drepanocytes (splenic sequestration). (Hematoxylin and eosin stained) (Original magnification, ×10 in A, ×40 in B and C, ×60 in D.)
